# The Causes and Potential Injurious Effects of Elevated Serum Leptin Levels in Chronic Kidney Disease Patients

**DOI:** 10.3390/ijms22094685

**Published:** 2021-04-28

**Authors:** Justyna Korczynska, Aleksandra Czumaj, Michal Chmielewski, Julian Swierczynski, Tomasz Sledzinski

**Affiliations:** 1 Department of Pharmaceutical Biochemistry, Faculty of Pharmacy, Medical University of Gdansk, 80-211 Gdansk, Poland; justyna.korczynska@gumed.edu.pl (J.K.); aleksandra.czumaj@gumed.edu.pl (A.C.); 2 Department of Nephrology, Transplantology and Internal Medicine, Faculty of Medicine, Medical University of Gdansk, 80-211 Gdansk, Poland; michal.chmielewski@gumed.edu.pl; 3 State School of Higher Vocational Education in Koszalin, 75-582 Koszalin, Poland; juls@gumed.edu.pl

**Keywords:** leptin, chronic kidney disease, cardiovascular risk, adipose tissue

## Abstract

Leptin is an adipokine that regulates appetite and body mass and has many other pleiotropic functions, including regulating kidney function. Increased evidence shows that chronic kidney disease (CKD) is associated with hyperleptinemia, but the reasons for this phenomenon are not fully understood. In this review, we focused on potential causes of hyperleptinemia in patients with CKD and the effects of elevated serum leptin levels on patient kidney function and cardiovascular risk. The available data indicate that the increased concentration of leptin in the blood of CKD patients may result from both decreased leptin elimination from the circulation by the kidneys (due to renal dysfunction) and increased leptin production by the adipose tissue. The overproduction of leptin by the adipose tissue could result from: (a) hyperinsulinemia; (b) chronic inflammation; and (c) significant lipid disturbances in CKD patients. Elevated leptin in CKD patients may further deteriorate kidney function and lead to increased cardiovascular risk.

## 1. Introduction

Chronic kidney disease (CKD) is a major clinical and public health problem. CKD can lead to end-stage renal disease (ESRD) and is a strong risk factor for cardiovascular morbidity and mortality [1]. The prevalence of CKD has increased in the last three decades, and it is now estimated that CKD affects more than 10% of the population worldwide [2]. Kidney Disease: Improving Global Outcomes (KDIGO), an organization that develops clinical practice for kidney diseases, defines CKD as the presence of structural or functional abnormalities in the kidneys for more than three months. The estimated glomerular filtration rate (eGFR) and urinary albumin excretion rate are biomarkers that are used to classify the stage of CKD, which is associated with the risk of disease progression, morbidity and mortality. Numerous studies have demonstrated deteriorations in patient prognosis with consecutive stages of CKD [3].

Adipose tissue is responsible for the secretion of many signaling molecules, including adipokines, hormones, cytokines and growth factors, such as leptin, adiponectin, resistin, tumor necrosis factor-α (TNF-α), interleukin 6 (IL-6), monocyte chemoattractant protein-1 (MCP-1), transforming growth factor-β (TGF-β), and angiotensin II [4,5,6]. Many of these factors play important roles in cardiovascular risk. Leptin is positively associated with cardiovascular risk, whereas adiponectin shows an opposite relationship [7]. ’Recently, it was reported that adiponectin is independently related to lower risk of coronary artery stenosis [8]. Adipokines can act both locally and systemically and can play significant roles in the pathogenesis of many diseases, such as obesity, diabetes, hyperlipidemia, hypertension, and atherosclerosis [9,10]. Patients with CKD often show abnormalities in adipokine profiles, which can potentially lead to increased inflammation, decreased appetite, protein-energy wasting (PEW) and the development of atherosclerosis [11,12,13,14]. One of the most investigated adipokines is leptin. Circulating leptin levels are elevated even in the early stages of CKD [7], and leptin levels increase with disease progression [15,16]. A number of studies have shown that leptin is involved in both CKD progression and CKD complications [17,18].

## 2. Leptin—Chemical Structure, Biosynthesis, Secretion and Functions

Leptin is a 167 amino acid protein adipokine with a molecular mass of approximately 16 kDa that is encoded by the LEP gene [19]. The sites of leptin synthesis are mainly but not exclusively adipocytes [20]. In humans, circulating leptin comes primarily from visceral and subcutaneous adipose tissue [21,22]. Studies have shown that both LEP expression levels in adipose tissue and serum leptin concentrations are increased in obese individuals [23,24]. Additional data indicate that circulating leptin levels correlate more strongly with the total mass of adipose tissue than with body mass index (BMI) [25]. The synthesis and secretion of leptin in adipose tissue can also be modulated by many factors, including sex, hormones and drugs. For example, glucocorticosteroids, insulin, estrogens, IL-6 and c-reactive protein (CRP) lead to increased levels of leptin, while starvation, metabolic acidosis, androgens, exposure to cold, agonists of β-adrenergic receptors, TNFα, growth hormone, somatostatin and smoking inhibit the production of leptin [14,26,27,28]. At a relatively low level, LEP is also expressed in the gastric mucosa, pituitary gland, hypothalamus, placenta, skeletal muscles, bone marrow, breast epithelium, ovaries, liver and brown adipose tissue [4,28,29,30].

Leptin is a pleiotropic adipokine. The main physiological role of leptin is to transmit information to the hypothalamus about the amount of stored energy, such as the mass of adipose tissue, and to influence energy expenditure by reducing appetite [4,31]. However, it has been shown that the role of leptin is highly complex and extends beyond controlling nutritional behavior and energy balance. Leptin is involved in the regulation of immune and neuroendocrine functions, sexual maturation, and reproductive functions. Leptin also affects carbohydrate and lipid metabolism [32,33,34], bone mass, inflammation, blood pressure, angiogenesis, hematopoiesis, and the stress response, and has a natriuretic effect on the kidney [4,25,35,36,37]. Circulating leptin reaches target organs, where it binds to specific receptors (known as ObR, LR or LEPR) located on the cell surface. Leptin receptors have been identified in many organs, including the kidney, pancreatic islets, the liver, the spleen, skeletal muscle, bone marrow, ovaries, testes, the heart, and the lungs, and in macrophages [30]. There are five isoforms of the leptin receptor in humans (ObRa, ObRb, ObRc, ObRd, and ObRe) [12]. Of all isoforms, only the isoform ObRb (also known as the long isoform) is considered a fully active receptor because it is able to fully transduce an activation signal into the cell. This isoform is highly expressed in the central nervous system (CNS), especially in the hypothalamus, where it takes part in the regulation of secretory activity in this organ. Ob-Ra, a short isoform, is the most common receptor in peripheral tissue, including the kidney [28]. Depending on the site of action (organ/tissue) and the type of ObR, the binding of leptin to receptors can result in the activation of different signaling pathways. The effects of leptin are mediated through five major signaling pathways. These pathways include the JAK-STAT, PI3K, MAPK, AMPK, and mTOR signaling pathways [38]. In circulation, leptin exists as a free, monomeric hormone or binds to the soluble ObRe receptor isoform. There is a clear sexual dimorphism in leptin levels in the blood, in which circulating leptin levels are generally higher in women than in men. The normal concentration of leptin in women is 8.8 ± 2.1 ng/mL, and in men, it is 2.2 ± 0.3 ng/mL. However, serum leptin levels in healthy adults can range from 0.5 to 37.7 ng/mL in men and from 2.0 to 45.2 ng/mL in women [25]. The half-life of circulating leptin in a human was determined to be 20–30 min, and the kidney plays a vital role in the clearance of circulating leptin [39].

## 3. Effects of Kidney Function on Blood Leptin Levels in Patients with CKD

Due to its relatively small size, leptin freely passes through the glomerular filter of the kidneys and is then reabsorbed in the proximal part of the convoluted tubules. The basic mechanism of leptin reabsorption is receptor-mediated endocytosis [40]. The multiligand scavenger receptor megalin, which is a member of the superfamily of LDL receptors located on the apical membrane of tubular epithelial cells, is involved in this process [38,41]. Moreover, studies have suggested that leptin internalization is mediated by a different type of megalin than the one responsible for albumin endocytosis [42]. Leptin receptors, mainly the ObRb isoform, were found in the inner medulla and vascular structures of the corticomedullary area. Since *ob*/*ob* (genetically obese) and *db*/*db* (genetically diabetic) mice exhibit morphological and functional kidney abnormalities similar to those of human diabetic nephropathy, it has been suggested that leptin plays a functional role in the kidney and that the kidneys are not only responsible for removing leptin from the circulation but are also sites of action of this adipokine [9]. In the late 1990s, Heimburger et al. and Nordfors et al. suggested that the rate of leptin elimination from the circulation by the kidneys determines its serum level, so any disturbance in glomerular filtration in patients with moderate and advanced CKD results in elevated blood leptin levels. Most clinical studies also indicate increased leptin levels in the blood of CKD patients [43,44,45,46,47]. Moreover, the serum levels of leptin increase with declining GFR in CKD patients [48].

## 4. Effects of Leptin Gene Expression in Adipose Tissue on Blood Leptin Levels in CKD Patients

The inability of the kidney to remove leptin from the circulation is probably not the only mechanism responsible for the development of abnormal levels of this adipokine. It is very likely that the increased leptin levels in the blood of CKD patients result from leptin overproduction in the adipose tissue due to hyperinsulinemia and chronic inflammation (increased levels of CRP, IL-6, IL-10, and TNF-α) [16,27]. Moreover, Stenvinkel et al. demonstrated that hyperinsulinemia and insulin resistance may contribute to hyperleptinemia in ESRD patients [49]. Studies conducted in vitro and in animal models, as well as in humans, have shown that leptin secretion is regulated by proinflammatory cytokines such as TNF-α and IL-6 [16,50]. Heimbürger et al. and Nordfors et al. [43,44] have shown that an increased level of CRP causes an increase in leptin levels. In CKD patients, there is a high correlation between serum leptin concentration and the amount of adipose tissue [51]. This relation also suggests the importance of leptin production in the adipose tissue of CKD patients for their leptin concentrations in the blood.

Additionally, the fact that the leptin gene polymorphism is associated with the risk of CKD [52,53] suggests the importance of leptin gene expression for CKD development. Genome-wide association studies are widely used to pinpoint specific single nucleotide polymorphisms (SNPs) to specific traits/diseases. In the case of leptin, most data focus on the relationship between SNP in leptin or leptin receptor genes and circulating leptin levels [54,55,56]. However, genome-wide associations between *LEP* and CKD are poorly studied. To the authors’ best knowledge, only two papers discuss the potential link between CKD and *LEP.* SNP 2548G/A was proposed as a risk marker of CKD progression to ESRD. However, the study was conducted only on the Egyptian population [52]. Another genetic variation in the leptin gene (STS-U43653) may be associated with significantly higher eGFR in the Xhosa population (a Nguni ethnic group in Southern Africa). Both results were not yet confirmed in other populations or the general population [53]. Polymorphisms of leptin and leptin receptor genes were also associated with the risk of cardiovascular disease in European and Asian populations [57,58,59,60]. However, none of the studies distinguished a CKD subgroup.

Aminzadeh et al. and Kalbacher et al. demonstrated that incubating murine 3T3-L1 adipocytes in a medium containing plasma from hemodialysis patients or patients with ESRD could induce the release of significantly more leptin by adipocytes than medium containing plasma from healthy subjects [50,61]. Thus, one can conclude that some molecules from patient plasma induce leptin production and release from adipocytes. Our earlier studies revealed decreased leptin mRNA levels in the adipose tissue and normoleptinemia in rats with kidney injury [62]. However, our recent study showed significantly increased expression of the *LEP* gene in subcutaneous adipose tissue from CKD patients compared to the controls. Moreover, the adipose tissue level of leptin mRNA positively correlated with serum leptin concentration [47]. This finding suggests that increased leptin production by adipocytes may contribute to the hyperleptinemia observed in CKD. The question then arises: what is the factor affecting the changes in expression of the gene encoding leptin in the adipose tissue?

Significant lipid disturbances are observed in patients with CKD [47,63]. The most commonly reported changes involve the levels of triacylglycerol and cholesterol. However, some data indicated significant alterations in the fatty acid (FA) profile in this group of patients [64,65,66]. Alterations in the FA profile may potentially increase cardiovascular risk in CKD patients [67]. Changes in the lipid profile may actively contribute to the deterioration of kidney function and disease progression [63,68]. However, not all consequences of these alterations are well understood. Czumaj et al. reported that the altered FA profile in CKD patients was connected to increased expression of genes involved in lipid metabolism in hepatocytes [64]. A study on 3T3-L1 adipocytes incubated with FA isolated from the serum of CKD patients showed significant increases in leptin mRNA levels [47]. Therefore, the changes in the FA profile associated with the course of CKD may contribute to the increased concentration of leptin by increasing the expression of the *LEP* gene in adipose tissue [47].

**Figure 1:**
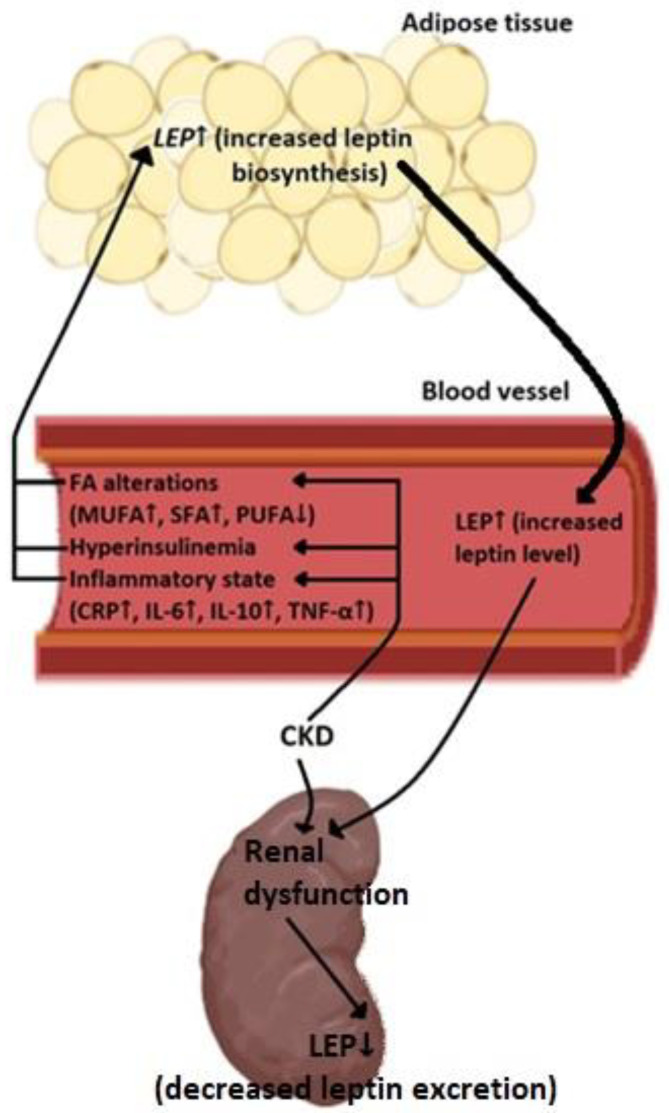
The potential mechanisms leading to hyperleptinemia in patients with CKD. SFA—saturated fatty acids, MUFA—monounsaturated fatty acids, PUFA—polyunsaturated fatty acids, CRP—C Reactive Protein, IL—interleukin, TNF-α—tumor necrosis factor α. The bold arrow symbolizes the increased secretion of leptin.

These results suggest that in CKD patients, (a) an increase in leptin synthesis takes place in the adipose tissue, which contributes to hyperleptinemia, and (b) chronic inflammation, hyperinsulinemia and alterations in the FA profile could be important factors that stimulate leptin synthesis in the adipose tissue (Figure 1).

Overall, the results presented so far suggest that in CKD patients: (a) the rate of leptin elimination from the circulation by the kidneys and (b) the stimulation of leptin biosynthesis in the adipose tissue both determine serum leptin level (Figure 1).

## 5. The Harmful Effects of Elevated Leptin Levels on Renal Function in CKD Patients

The main functions of the kidneys are the excretion of waste products, the regulation of water homeostasis and blood pressure, and the secretion of hormones (erythropoietin and 1,25(OH)_2_D_3_) and enzymes (e.g., renin). In healthy kidneys, only a small proportion of proteins (mainly albumin) passes through the glomerular filtration barrier, and even this load is reabsorbed in the proximal convoluted tubule, resulting in only trace amounts of albumin in urine. Leptin plays a role in disturbing this homeostasis.

Hyperleptinemia in CKD may promote pathophysiological changes in the kidney, leading to disease progression and comorbidity [18]. These pathological changes can occur in various types of cells, both within the renal glomeruli (endothelial cells, mesangial cells and podocytes) and in proximal tubules [28]. These changes may result in increased protein leakage into the filtrate, which is mainly driven by the thickening of the basement membrane, which surpasses the endocytosis capabilities of proximal tubular cells [69]. Wolf et al. described the direct profibrotic effects of leptin on glomeruli in in vivo and in vitro models and suggested the existence of a cross-talk between leptin and glomerular endothelial/mesangial cells [70,71]. Leptin stimulates the proliferation of renal glomerular endothelial cells and increases the expression of TGFβ1, a key mediator of fibrogenesis [16,28], in these cells (by binding to the ObR receptor, leptin activates the JAK/STAT signaling pathway). Increased leptin levels also contribute to the increased expression of type IV collagen in the kidney [9]. Leptin induces glomerular mesangial cell proliferation by activating the PI3K pathway. Mesangial cell hypertrophy increases the amount of filtered protein and albumin that reaches proximal tubule cells and, as a result, activates inflammatory pathways and fibrosis [31]. Moreover, an increase in the synthesis of TGFβ-1 receptor and type I collagen, as well as increased glucose uptake by mesangial cells, have been found [28]. TGFβ-1 secreted by endothelial cells acts in a paracrine manner on the mesangium by binding to its receptor and activating the synthesis of extracellular matrix (ECM) proteins, including collagen, fibronectin, tenazine and proteoglycans. Therefore, an increased level of TGFβ-1 leads to the accumulation of ECM and consequently to glomerular fibrosis and glomerulosclerosis [16]. In podocytes, leptin contributes to decreased expression of the proteins responsible for proper glomerular filtration, including podocin, nephrin, podoplanin, and podocalyxin [18]. In proximal convoluted tubule cells (PTCs), leptin reduces the metabolic activity of cells by activating the mTOR signaling pathway. Reductions in protein levels in cells have also been observed; however, the molecular mechanism of this process remains unknown [23,72]. Furthermore, leptin, via the AMPK-mediated pathway, can upregulate TGF-β1, reduce megalin, and reduce albumin processing in PTCs [69]. Leptin appears to activate signaling pathways in the renal tubules by binding to megalin and in mesangial cells through the ObR receptor [72]. Leptin also mediates the activation of the sympathetic nervous system (SNS) in the kidney and the renin–angiotensin–aldosterone system. Both increased SNS activation and direct renal effects leading to sodium retention may explain the increased blood pressure associated with chronic hyperleptinemia [28,73,74].

CKD is characterized by increased oxidative stress and inflammation. Leptin can activate NADPH oxidase and generate reactive oxygen species (ROS), which affect the structure and function of the kidneys [9]. ROS-induced activation of transcription factors and proinflammatory genes exacerbates existing inflammation. ROS disrupt the excretory function of the nephron, lead to the accumulation of metabolic products and impair renal regulatory mechanisms [75]. Importantly, both oxidative stress and inflammation are critical components of CKD-related pathologies that can negatively affect the entire body, leading to many disorders [27,76,77]. The results of the Olivetti Heart Study confirmed the key role of leptin in the development of renal dysfunction. High circulating leptin levels may predict an increased risk of renal function loss with age in healthy adult men [78]. Obesity is associated with hyperleptinemia [4], and is a major independent risk factor for CKD [74,79]. Thus, one may suppose that hyperleptinemia associated with obesity contributes to the development of renal dysfunction in obese hyper-leptinemic patients.

**Figure 2:**
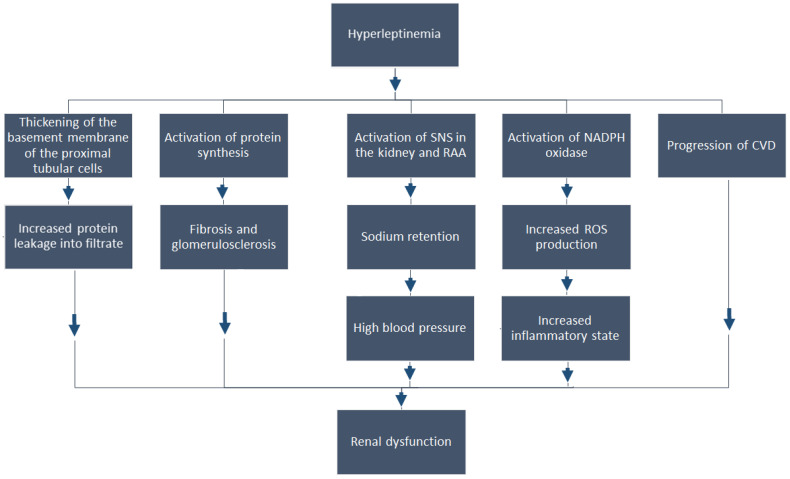
The potential mechanisms of the harmful effect of hyperleptinemia on kidney function in CKD patients. CVD—cardiovascular disease, ECM—extracellular matrix, RAA—renin–angiotensin–aldosterone system, ROS—reactive oxygen species, SNS—sympathetic nervous system.

The results discussed in this section suggest that the harmful effect of hyperleptinemia on CKD patients could be associated with (a) an increase in protein leakage into the filtrate; (b) the accumulation of ECM, glomerular fibrosis and glomerulosclerosis; and (c) an increase in oxidative stress and inflammation (Figure 2).

## 6. Effects of Increased Leptin Concentrations on the Risk of Cardiovascular Disease in Patients with CKD

Numerous studies have shown that a decrease in eGFR is associated with increased frequency and severity of cardiovascular disease (CVD) [14]. CKD, together with hypertension and diabetes, is one of the most common causes of CVD regardless of other conventional risk factors. CKD and ESRD pose a 5 to 10 times greater risk of developing CVD in comparison to that of a control group of similar age [75]. Moreover, non-traditional risk factors such as endothelial dysfunction, vascular calcification, volume overload, oxidative stress, and inflammation play important roles in the development of CVD in CKD patients. Inflammation and oxidative stress have recently gained considerable attention as being relevant to the initiation of CVD during the course of CKD [75]. Moreover, they contribute to albuminuria and/or proteinuria, which themselves predict CVD in the course of CKD [80,81]. Potential mechanisms of the increased cardiovascular risk in CKD also include changes in the lipid profile and serum adipokine levels. Over the past few years, changes in adipokine levels in the context of CKD progression and the risk of comorbidities have been investigated by many researchers [9,13,14,15,27,76,82,83].

Leptin may contribute to the development of cardiovascular dysfunction by inducing inflammation, oxidative stress and endothelial dysfunction [18,76]. It is well known that hyperleptinemia is associated with vascular smooth muscle cell hypertrophy. Leptin induces the proliferation of endothelial cells, promotes angiogenesis by influencing the level of VEGF, and increases platelet aggregation. The proatherogenic role of leptin is enhanced by the induction of inflammation [16]. Moreover, studies have shown that in patients with advanced CKD, leptin levels are positively correlated with aortic stiffness [84].

Vascular endothelial dysfunction is an essential factor that precedes and facilitates the development of atherosclerosis. These disorders are common in the course of CKD [85]. Accumulating evidence from both in vivo and in vitro studies link circulating leptin levels with endothelial dysfunction (ED) [86]. In patients with CKD, altered serum leptin levels are associated with increased levels of ED markers, including soluble endothelial adhesion molecules (sICAM-1 and sVCAM-1). sICAM-1 and sVCAM-1 are inflammatory factors that influence endothelial remodeling. In in vitro studies with HUVEC cells, leptin activated the Akt/GSKβ/β-catenin pathway, leading to increases in ICAM-1 and VCAM-1 levels and the rearrangement of the cytoskeleton. This, in turn, resulted in increased migration of endothelial cells and increased monolayer permeability, which ultimately resulted in ED [9,87]. Moreover, HUVECs supplemented with leptin showed increased expression of proinflammatory factors, such as IL-6, ET-1, and MCP-1, and as a consequence of these changes, f-actin recombination, vinculin aggregation, and endothelial cell migration were observed [86]. There is increasing evidence that leptin can induce mitosis and determine cell survival via activation of the Wnt/β-catenin signaling pathway, which is inactive in healthy kidneys. In vivo studies have shown that leptin increases Wnt1 expression and β-catenin accumulation. This pathway may, therefore, be a major link between ED and CKD [86].

The aforementioned oxidative stress caused by leptin may also contribute to the dysfunction of blood vessels and promote the development of atherosclerosis by modifying lipid components, lipoproteins, and proteins. ROS affect the progression of atherosclerosis by reducing the availability of nitric oxide (NO), which is necessary to maintain proper vessel functions. Altered NO metabolism in CKD can be an important element linking oxidative stress with atherosclerosis [75,76,77]. Thus, high levels of leptin may also affect vascular endothelial cells and contribute to the initiation and progression of CVD in patients with CKD.

## 7. Conclusions

Many lines of evidence suggest an association between leptin and CKD. It seems that leptin is involved in both disease development and the development of CKD comorbidities. The molecular mechanism (a) leads to elevations in serum leptin concentrations in CKD patients, and (b) harmful effects of hyperleptinemia on kidney function and associated diseases seem to be complex and need to be further explained. Future studies are needed to determine whether patients with CKD would benefit from therapeutic modulation of circulating leptin levels.
